# Is the *Angiostrongylus vasorum* infection in domestic dogs underestimated or misdiagnosed? A comprehensive presentation of four lethal cases

**DOI:** 10.3389/fvets.2023.1146713

**Published:** 2023-05-18

**Authors:** Marian Aurel Taulescu, Andrada Negoescu, Andrei Ungur, Corina Toma, Angela Monica Ionică, Claudiu Gal, Ioana Sandu, Alexandru Buzdea, Andrei Tutuneanu, Mihai Turcitu, Ioan Emilian Horvat, Georgiana Deak

**Affiliations:** ^1^Department of Anatomic Pathology, University of Agricultural Sciences and Veterinary Medicine, Cluj-Napoca, Romania; ^2^Synevovet, Bucharest, Romania; ^3^Clinical Hospital of Infectious Diseases of Cluj-Napoca, Cluj-Napoca, Romania; ^4^Department of Parasitology and Parasitic Diseases, University of Agricultural Sciences and Veterinary Medicine, Cluj-Napoca, Romania; ^5^Pet Stuff Veterinary Hospital, Bucharest, Romania; ^6^Omnivet Impex, Bucharest, Romania; ^7^Best Romvets, Alba-Iulia, Romania

**Keywords:** *Angiostrongylus vasorum*, cardiopulmonary disorder, diagnosis, dogs, lungworms, pathology, Romania

## Abstract

**Introduction:**

*Angiostrongylus vasorum* (*A. vasorum*) is a widely distributed gastropod-borne nematode, causing severe cardio-pulmonary disorders in dogs. In Romania, *A. vasorum* was detected in foxes and serologically confirmed in domestic dogs, but no clinical cases are currently diagnosed.

**Methods:**

Four dogs with clinical history of respiratory distress, originating from different geographical regions of Romania, were included in the study. One dog (case 1) was clinically evaluated using thoracic radiology and cardiac ultrasound; examination of feces and PCR were also performed for the etiologic diagnosis. The postmortem exam was performed in the other three cases, followed by parasitological and molecular analyses.

**Results:**

In the first case, parasitic pneumonia was suspected based on the radiographic examination of the thorax and the infection with *A. vasorum* was confirmed by L1 morphological identification and PCR. The main postmortem changes included large, coalescing, dark red areas of pulmonary consolidation (*n* = 3) and numerous adult nematodes in the pulmonary arteries (*n* = 2). The histopathological examination of the lungs showed necrotizing and granulomatous pneumonia with severe hemorrhages and chronic pulmonary arterial changes. Intralesional nematodes were seen in all necropsied cases. Additional inflammatory changes related to *A. vasorum* infection were identified in the brain and tracheobronchial and mediastinal lymph nodes (*n* = 2). Identification of larvae, histopathology and PCR confirmed the infection with *A. vasorum*.

**Conclusions:**

This study describes the first cases of canine cardiopulmonary angiostrongylosis in domestic dogs in Romania, and focuses on clinical presentation, pathological findings and molecular analysis. Angiostrongylosis should be included on the list of differential diagnoses of canine cardiopulmonary distress and/or haemorrhagic diathesis in Romania and awareness should be raised among clinicians to avoid post-mortem diagnosis in the future.

## 1. Introduction

*Angiostrongylus vasorum* (Nematoda: Metastrongyloidea) is a cardiovascular nematode, that resides in the pulmonary arteries and the right heart of domestic dogs and other carnivores (1). The biological life cycle is indirect with an intermediate obligatory host represented by terrestrial or aquatic gastropods, and may include paratenic hosts (frogs and domestic chickens) infected with the third larval stage (L3) (2). The natural definitive host is the red fox (*Vulpes vulpes*), an important reservoir that contributes to bridging infections in domestic animals (3, 4).

Infected dogs can develop severe thrombotic pulmonary endarteritis and pneumonia with varying clinical signs, ranging from minor respiratory distress to severe manifestations with a fatal outcome (5, 6). Respiratory symptoms (e.g., coughing, dyspnea), and exercise intolerance are caused by first stage larvae (L1) that migrate in the lungs (7, 8). In some cases, the lung pathology may progress to cor pulmonale associated with extrapulmonary lesions and clinical signs (9).

Clinical signs related to coagulation disorders were observed in some dogs infected with *A. vasorum*, and included hematochezia and haemorrhagic diathesis (5, 8).

Atypical clinical manifestations are produced by the larval migration in various organs or even adult stages in the eye, with panuveitis with hyphema, photophobia, and corneal edema (10).

Difficulties in making the specific diagnosis can arise when an animal exhibits non-specific symptoms such as weight loss, anorexia, lethargy or fever (8).

Neurological signs represent almost 4% of the clinical manifestations and, they can vary from ataxia, paresis, paralysis or seizures (5). Both hemorrhages due to coagulopathy (11) and larval migration (12) may cause neurological manifestations in dogs infected with *A. vasorum*.

Several diagnostic methods are available that can be used for the detection of canine angiostrongylosis. Until present, the “gold standard” method remains the modified Baermann technique for the detection of the L1 passed in the feces. Other tests like serological detection of circulating antigens (13, 14), or antibodies against *A. vasorum* (15), or isolating the parasites' DNA from different substrates (16, 17) are also available.

Since the first description in Toulouse (France) (18), this nematode gained a lot of interest among European parasitologists, mainly due to its broad and severe clinical picture. Two decades ago, the disease was sporadically reported in some parts of the United Kingdom, Denmark, France, Italy, and Spain (7). More recently, a sudden spread of the disease was observed in different locations in the same countries and in completely new geographical areas. Most likely, this expansion is correlated to the extended distribution of the red fox population (19).

Currently canine angiostrongylosis is an emerging disease reported in almost all European countries (1). Autochthonous infections by *A. vasorum* were identified in other parts of the world with three new different geographical regions reported in the last year (20–22).

In Romania, infection by *A. vasorum* was first confirmed in red foxes in 2017 (23) and 2 years later it was serologically confirmed also in domestic dogs (24). More recently, a nationwide survey among Romanian veterinarians proved that although still new for the country, *A. vasorum* infections were diagnosed in domestic dogs in different locations (25). However, one of the veterinarians that admitted diagnosing a case, treated it with melarsomine, a nematicide used for the treatment of *Dirofilaria immitis* (25).

Considering the known presence of the “French heartworm” in Romania, the severity of the disease and the challenges in diagnosing it, this study aimed to describe four lethal cases of canine angiostrongylosis, focusing on the clinical aspects and pathological findings, and to raise awareness among Romanian veterinarians.

To the best of our knowledge, no other clinical cases of canine angiostrongylosis have been previously reported in Romania.

## 2. Material and methods

### 2.1. Clinical history

Clinical signs were reported for all dogs and are summarized in Table 1.

**Table 1:** Signalment and clinical presentation of dogs infected with *A. vasorum*.

**Case**	**Breed**	**Age**	**Sex**	**Clinical signs**
Case 1	Yorkshire terrier	10 years	M	Chronic dyspnea, coughing, low resistance to effort, cyanotic mucous membranes, crackling noise in lung area, grade II/VI systolic murmur
Case 2	Caucasian shepherd	8 years	F	Chronic dyspnea, coughing, low resistance to effort, apathy, vomiting
Case 3	Poodle	1.5 year	F	Acute dyspnea, coughing
Case 4	Mixed breed	5 months	F	Chronic dyspnea, coughing, apathy, anemia

#### 2.1.1. Case 1

A 10-year-old, intact male, Yorkshire terrier dog was referred to a private veterinary hospital from Bucharest (Pet Stuff Veterinary Hospital) on January 2021 with coughing, chronic dyspnea and cyanotic mucous membranes. The dog was previously diagnosed with degenerative mitral valve disease (stage B2) and pulmonary hypertension, for which it has received medication, but no deworming was performed within the last year. The dog was living in the periphery of the city, in a house with yard, near a lake. It was the only pet of the family and had no travel history. Upon physical examination, the animal showed increased respiratory rate, exercise intolerance, crackling noise in the pulmonary area, and obtunded mentation, besides abnormal grade II/VI systolic murmur. Point-of-care lung ultrasound (LUS) examination revealed numerous multifocal B-lines. Complete blood count (CBC) report showed marked reticulocytosis [205.2 K/μl reference interval (RI): 10.0–110.0] without evidence of anemia, most likely physiologic reticulocytosis (Hct: 43.0% RI: 37.3–61.7), inflammatory leukogram with a total white cell count of 18.57 K/μL (RI: 5.05–16.76) and neutrophilia with regenerative left-shift (14.01 K/μL, RI: 2.95 - 11.64), mild monocytosis (1.63 K/μL RI:0.16–1.12) suggesting stress leukogram and inflammatory response and non-reactive thrombocytosis (747 K/μL, RI:148–484) associated with inflammation and a redistribution mechanism. Routine serum biochemical profile revealed mild hyperglycemia, most likely stress induced (138 mg/dL; RI: 60–116 mg/dL), while lactate, blood urea nitrogen, creatinine, and electrolytes (sodium, potassium, chloride, calcium) were within normal limit values. The coagulation profile showed a prolonged prothrombin time (PT) (24.4 s; RI: 14–20 s) but normal activated partial thromboplastin time (aPTT) (120.1 s; RI: 94–123 s). A slightly increased PT with normal aPTT can suggest a disseminated intravascular coagulation (DIC) status. Quantitative D-dimer concentration was within reference intervals (0.27 μg/ml; RI: 0.023–0.65 μg/ml).

**Figure 1:**
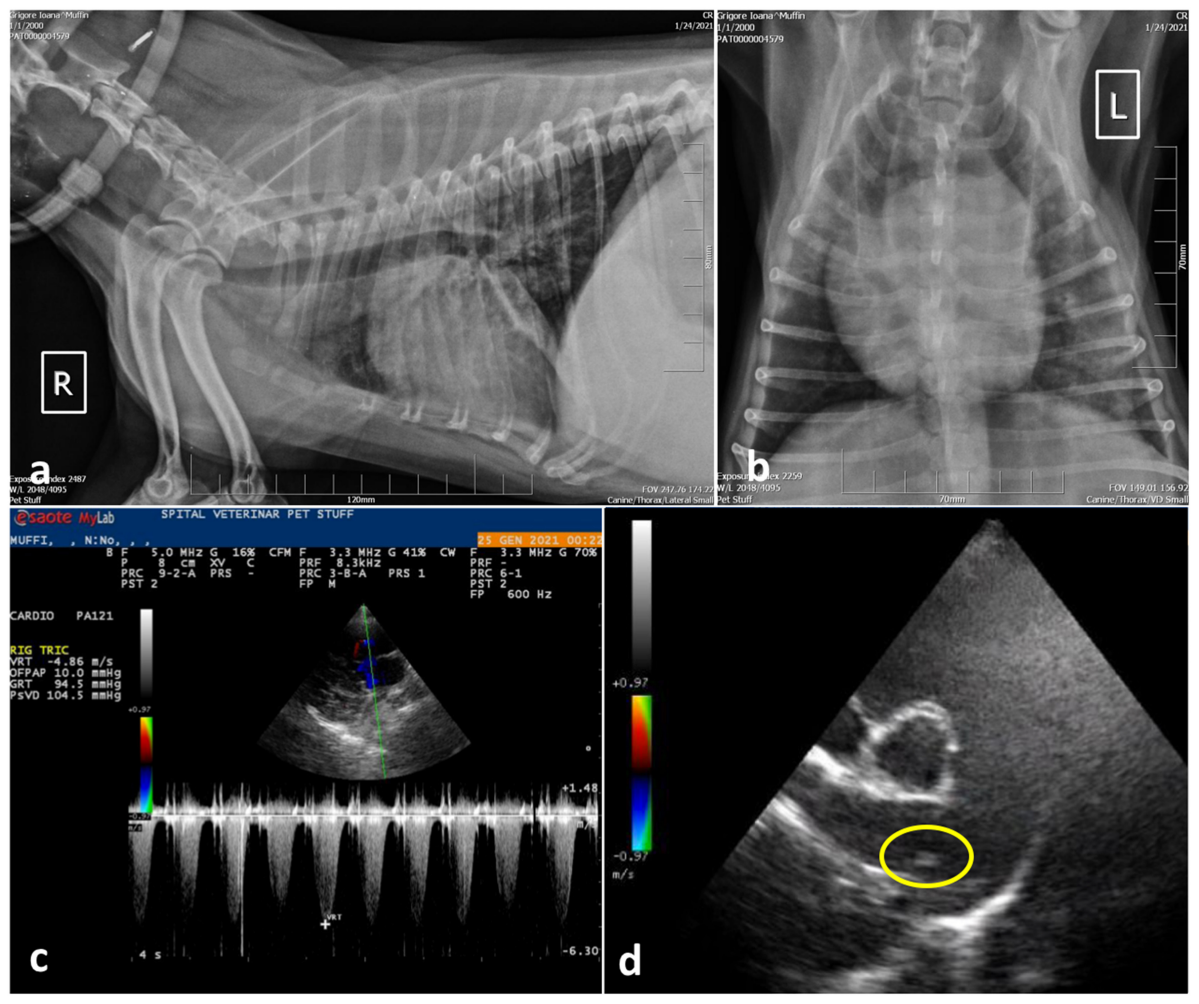
Case 1, thoracic radiographs: **(a)** Note the small distribution of areas with diffuse interstitial pulmonary pattern and pronounced peribronchial infiltration around the large bronchi; **(b)** Ventro-dorsal projection of the thorax radiography showing marked cardiomegaly with right ventricular enlargement; **(c)** Spectral Doppler study using continues wave revealed tricuspid regurgitant (TR) flow with a peak velocity >4.8 m/s suggesting severe PH (PG > 90 mmHg) and; **(d)** Right parasternal short axis view, slightly oblique for pulmonary trunk and right pulmonary artery optimal visualization. The main pulmonary artery is distended and parallel echogenic lines separated by a hypoechoic region were observed in the right main pulmonary artery (encircled area), suggesting a tubular structure, most probably the worm profile.

Following initial stabilization, a radiographic examination of the thorax and cardiac point of care ultrasound (POCUS) were performed. Radiology revealed small areas of interstitial pulmonary pattern and pronounced peribronchial infiltration around the large bronchi (Figures 1a, b). Parasitic pneumonia was suspected and a broncho-alveolar lavage was recommended but declined by the owner.

The cardiac POCUS images were reviewed by the cardiologist and showed signs of severe pulmonary hypertension (PH) with right ventricular enlargement, systolic flattening of the interventricular septum, the pulmonary artery to aorta ratio >1.0 and a peak tricuspid regurgitation velocity of ~4.86 m/s, PG 94 mmHg. In addition, parallel echogenic lines separated by a hypoechoic region were observed in the right main pulmonary artery in transverse and longitudinal views (right parasternal long axis 5 chambers and right parasternal short axis heart base) (Figures 1c, d). Type 5 PH was diagnosed, and further heartworm (*Dirofilaria immitis*) testing was performed by collecting a sample of peripheral blood, which was tested directly by means of semiquantitative PCR, and serologically by means of a rapid test (Idexx SNAP 4Dx Plus), respectively. Both exams came negative.

A sample of feces was tested using the Baermann-modified technique (26). Several collected larvae (*n* = 9) were morphologically identified as *A. vasorum* L1 based on their specific morphology and subsequently confirmed by PCR.

The dog was treated with a single dose of imidacloprid 10%, moxidectin 2.5% spot-on solution (Advocate; Bayer AG) in the clinic and the owner was advised to repeat the treatment at 4 weeks and to refer to a cardiologist but denied any other medical procedures or treatment. Four weeks after the initial treatment, via telephonic follow up the dog was better. The dog died 6 months after the initial diagnosis due to severe respiratory distress. A postmortem examination was not performed.

#### 2.1.2. Case 2

The second case was an 8-year-old, intact female Caucasian shepherd dog from Cipău village (Mure? County). The dog was bought as a puppy at 4 months of age from Turda (Cluj county), and kept in the mentioned location since then. A representative part of Cipău village, 6.000 m^2^ (out of 10.000 m^2^) are fishing lakes populated with different fish species.

The dog showed low resistance to effort, chronic respiratory distress (dyspnea, coughing) and vomiting, and became more apathetic with each day. It was treated for pneumonia with different antibiotics (Fluoroquinolone, Doxycycline, and Amoxicillin-Clavulanic Acid) over a period of 10 months, without improvement. The clinical signs aggravated after ~10 days after weaning the puppies. The dog suddenly died 1 month later. The owners mentioned that the dog used to eat much dead or alive fish from the lakes. The last deworming was performed more than 6 months before the moment of death. There was another dog in the same household, which was tested using the modified Baermann funnel technique after the diagnosis was confirmed in the first animal, with negative results. The second dog did not consume fish.

#### 2.1.3. Case 3

The third dog was a 1.5-year-old, intact female poodle, originating from Alba-Iulia (Alba County). The bitch developed acute dyspnea and coughing, and died after 20 min after the initial consult. There were other two mixed-breed dogs in the same household, which were tested by larval concentration with negative results.

#### 2.1.4. Case 4

The fourth dog was a 5-month-old, intact female mixed breed originating from Reghin (Mure? county). The dog was referred for apathy, anemia and chronic respiratory distress (dyspnea, coughing) to a private veterinary clinic and died 1 h after the initial examination.

### 2.2. Postmortem examination

During postmortem examination, the pulmonary arteries were isolated, longitudinally opened and examined from the base of the heart to the narrowest branches at the margins of all lung lobes and scrutinized for parasites (cases 2 and 4) (9). Detected nematodes were collected and stored in 4% formalin-labeled tubes for morphological identification and absolute ethanol for molecular analysis. Samples from the lungs, tracheobronchial and mediastinal lymph nodes, liver, kidneys, gastric mucosa and brain (cerebral cortex, cerebellum, thalamus, brainstem) were collected for cytological, and histological analyses. In case 3, the necropsy was performed by the clinician and lung tissue samples were submitted to the Department of Anatomic Pathology.

### 2.3. Cytology

For cytological analysis, the imprints from tracheobronchial lymph nodes and lungs (cases 2 and 4) were stained by Dia Quick-Panoptic (DQP, Reagent Ltd., Budapest, Hungary).

### 2.4. Histopathology

For histology, tissue samples collected from the cases 2, 3 and 4 were fixed in 10 % phosphate-buffered formalin (pH = 7.0) for 24 h, routinely processed, embedded in paraffin wax, cut into 2–3 μm sections, and stained with hematoxylin and eosin (H&E). Samples were examined using an Olympus BX51 microscope. Photomicrographs and measurements of the parasites for morphologic identification were taken using an Olympus SP 350 digital camera and Cell^*B*^ basic imaging software (Olympus Corporation, Japan).

### 2.5. Parasitological examinations

The adult nematodes were collected from the pulmonary arteries (cases 2 and 4) and were cleaned in sterile saline (NaCl 0.9 %) solution. Several specimens were then microscopically examined in temporary mounts, clarified using lactophenol, and morphologically identified based on the specific descriptions (27).

For morphologic evaluation of the larvae (cases 2, 3, and 4), the remaining lung tissue samples were homogenized with saline solution on a magnetic stirrer, filtered and the solution was centrifuged in 50 ml corning tubes. One drop of Lugol solution was added to the sediment which was then examined.

### 2.6. DNA extraction and molecular identification

In the first case, DNA was extracted using the collected sediment containing larvae using a commercial kit (Qiagen, Germany). One ml of Baermann liquid was centrifuged at 5,000 rpm for 3 min and the supernatant was discarded. DNA purification was done with the QIAamp DNA Mini kit (Qiagen, Germany) was used according to the manufacturer's instructions, with elution of nucleic acids in 150 μl final volume.

PCR confirmation was performed using previously described primers and probe (121-bp product from ITS-2 region) (28), with minor modifications for best complementarity to all available sequences (data available upon request) and PerfeCta qPCR ToughMIx kit (Quantabio, USA) according to manufacturer's guidelines. Samples were loaded into a RotorGeneQ cycler (Qiagen, Germany) and PCR was performed: 2 min incubation at 95°C for polymerase activation and initial denaturation, followed by 40 PCR cycles of 95°C for 8 s and 60°C for 30 s with fluorescence data collection on green (FAM) channel.

In cases 2 and 4, genomic DNA was isolated from one nematode using a commercial kit (Isolate II Genomic DNA kit, meridian Bioscience, London, UK) according to the manufacturer's instructions. Subsequently, a fragment of the mitochondrial *cytochrome c oxidase* subunit 1 (*cox*1, ~700 bp) gene was amplified by conventional PCR, using the universal primers LCO1490/HCO2198, according to literature (29). The PCR product was excised from the gel, purified on a silica membrane spin column (Gel/PCR DNA Fragments Kit, Geneaid Biotech, Taiwan) and sequenced bidirectionally using an external service (performed by Macrogen Europe B.V., Amsterdam, The Netherlands). The sequences were assembled using Geneious software (biomatters LTD, New Zealand) and compared to other sequences available in the NCBI GenBank^®^ database by Basic Local Alignment Search Tool (BLAST) analysis.

## 3. Results

### 3.1. Postmortem examination

#### 3.1.1. Case 2

**Figure 2:**
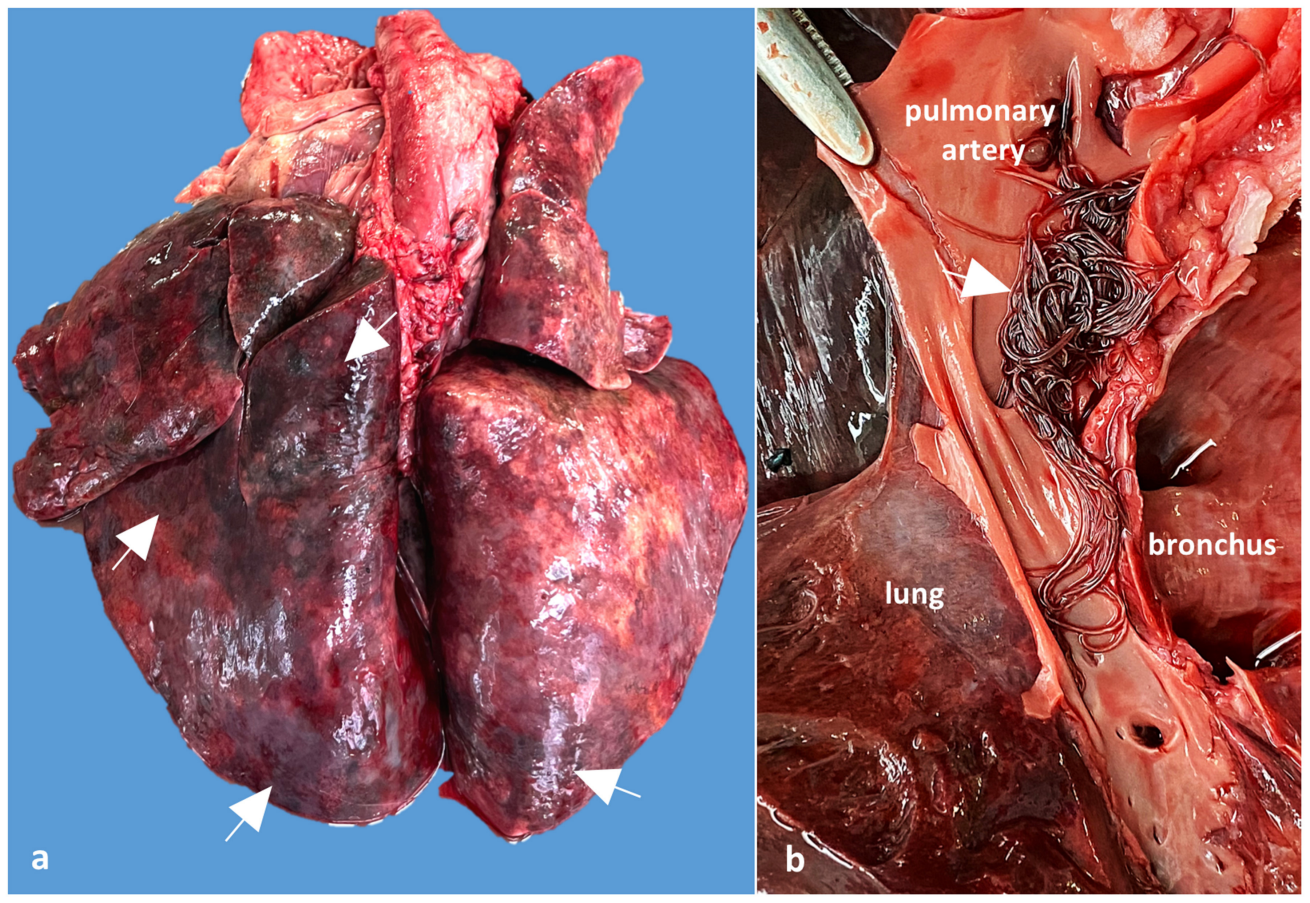
Case 2, Gross examination of the lungs and pulmonary arteries: **(a)** Lungs shows numerous dark red areas of densification, suggesting verminous pneumonia with necrosis and hemorrhages (arrows); **(b)** Detail of the opened pulmonary artery showing numerous adults of *A. vasorum* in the pulmonary artery (arrow).

The main postmortem changes consisted of severe cyanosis and bilateral pulmonary densification with multiple areas of hemorrhage and necrosis (cobblestone appearance) (Figure 2a). The pulmonary arteries and right ventricle contained numerous adult nematodes of *A. vasorum*, measuring between 2 and 2.5 cm in length (Figure 2b). A single adult female of *Dirofilaria immitis* was found in the right ventricle of the heart. Cardiomegaly with hypertrophy of the right ventricular wall because of chronic pulmonary lesions and hypertension (cor pulmonale) was also observed. The tracheobronchial lymph nodes were edematous and hemorrhagic. The gastric mucosa from the body region showed granular to round white-gray nodules, varying between 2 and 5 mm, interpreted as nodular gastritis. No other significant gross changes were noticed.

#### 3.1.2. Case 3

The clinician who performed the necropsy noted that the dog was in good body condition and the mucosae were pale. More than 100 ml of sero-hemorrhagic fluid was found within the thoracic cavity. The left lung failed to collapse and showed severe edema and multifocal dark red areas of densification due to necrosis and hemorrhages, affecting mostly the caudal lobe (Supplementary Figures 1a, b). The macroscopic evaluation of pulmonary arteries was not performed in this case.

#### 3.1.3. Case 4

The dog was in a good body condition and the main postmortem changes were represented by bilateral and multifocal to coalescing red areas of consolidation within the lungs (Supplementary Figure 2a). The pulmonary arteries contained numerous thread-like adult nematodes, measuring between 2 and 2.5 cm in length (Supplementary Figure 2b). Cardiomegaly with hypertrophy of the right ventricular wall and right atrial dilation was a significant change.

### 3.2. Cytology

In cases 2 and 4, Dia Quick-Panoptic stained imprints showed numerous larvae of *A. vasorum* in both lungs and tracheobronchial lymph nodes (Supplementary Figures 3a, b). In addition, a mixed inflammatory cell population, composed of macrophages, small lymphocytes, plasma cells and scattered neutrophils, and eosinophils were identified in the examined samples.

### 3.3. Histopathology

**Figure 3:**
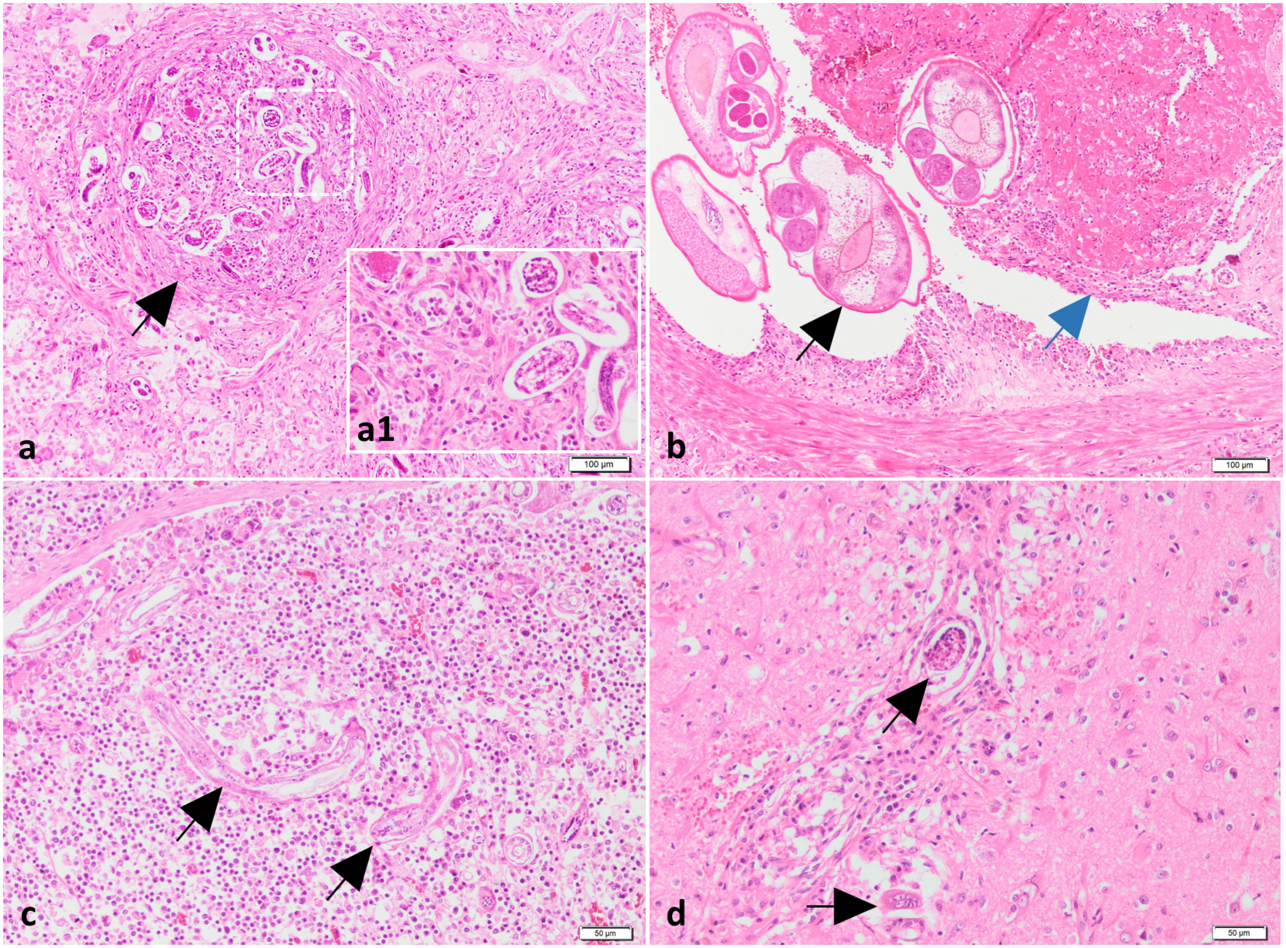
Histological findings of lungs, mediastinal lymph nodes and brain from case no. 2. **(a)** The lungs contain granulomas centered on nematode larvae and eggs (black arrow). H&E stain. Bar = 100 μm. Inset (higher magnification of figure **a**): nematode larvae and eggs surrounded by macrophages. **(b)** The pulmonary artery is partially occluded by a trombus (blue arrow) and numerous cross-sectioned adults of metastrongylid lungworms (black arrow). H&E stain. Bar = 100 μm. **(c, d)** Presence of parasites in the mediastinal lymph node (black arrow) **(c)** and neuroparenchyma (black arrows) **(d)** associated with a moderate inflammatory response. H&E stain. Bar = 50 μm.

Histologically, in all investigated cases (dogs 2, 3 and 4), the pulmonary parenchyma was multifocal to coalescing affected by variably-sized granulomatous nodules associated with hemorrhage, and centered on viable and fragmented parasitic organisms consisting of metastrongylid eggs and larvae (Figure 3a, a1). The granulomas were composed mainly of macrophages, multinucleated giant cells (foreign body and Langhans types), lymphocytes, plasma cells, and small numbers of eosinophils and neutrophils. The inflammatory nodules were occasionally rimmed by a thin fibrovascular tissue. The nematode eggs were thin-walled, ovoid, 50–70 μm in diameter, and contained a coarsely granulated basophilic material (morula) or a larva. The larvae were elongated and showed a thin acidophilic cuticle and a primitive intestinal tract. In some sections, the pulmonary arteries were markedly dilated and partially occluded by a thrombus, containing numerous longitudinal and cross sections of adult nematodes (Figure 3b). These parasites showed an eosinophilic cuticle, polymyarian-coelomyarian musculature, intestinal tract lined by multinucleated cells, and reproductive tract (Figure 3b). The wall of the affected arteries was severely and circumferentially thickened due to hypertrophy and hyperplasia of smooth muscle cells. The tunica intima was also thickened by fibrous tissue and inflammatory cells, represented by small lymphocytes, macrophages, and plasma cells. In other sections, the arterial lumen was occluded with organized thrombi, containing numerous blood-filled channels, fibroblasts and a few irregular collagen fibers (recanalization). The adjacent parenchyma was severely affected by coagulative necrosis associated with hemorrhage and neutrophilic infiltrates (pulmonary infarcts). Based on gross, cytological and histological findings a diagnosis of pulmonary angiostrongylosis was made.

The tracheobronchial and mediastinal lymph nodes were diffusely enlarged due to lymphoid hyperplasia and interfollicular and paracortical infiltration with macrophages, plasma cells and eosinophils. Nematode larvae were found in the subcapsular sinuses and cortex (Figure 3c). The cerebral cortex was multifocally affected by necrosis, vacuolation, hemorrhage, perivascular cuffs composed of lymphocytes, macrophages and neutrophils, and intralesional fragments of nematode larvae (cases 2 and 4) (Figure 3d). Moderate lymphoplasmacytic and eosinophilic gastritis with lymphoid follicle hyperplasia and mild colonization with *Helicobacter*-like organisms was found in the body region of the second dog. No significant changes were identified in the liver, kidneys and intestine of the investigated dogs.

### 3.4. Parasitological examinations

**Figure 4:**
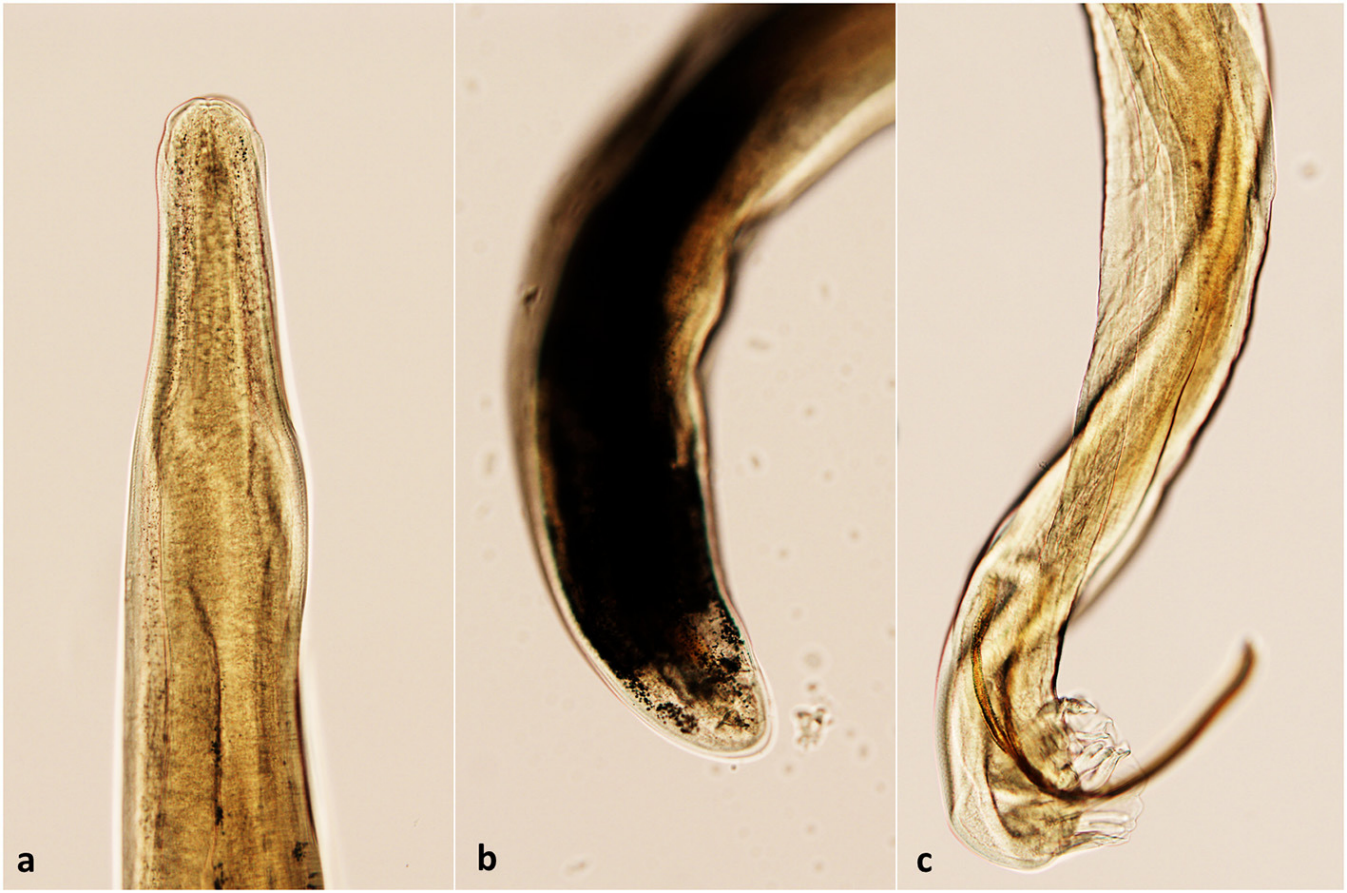
The morphological aspect of *A. vasorum* adult nematodes. **(a)** The anterior end (cephalic extremity); **(b)** Posterior end of a female *A. vasorum*; **(c)** Posterior end of a male *A. vasorum* with a short copulatory bursa and well-developed rays.

The obtained L1 were 330 to 360 μm in length with a non-rhabditiform esophagus, a terminal oral-opening and a waved tail with a dorsal spine and a notch consistent with the typical morphology of *A. vasorum* L1. All the examined adult nematodes collected from cases 2 and 4 were identified as *A. vasorum* (Figure 4).

### 3.5. Molecular analysis

A specific amplification curve was obtained in case 1, demonstrating *A. vasorum* DNA in the analyzed sample. The Ct value obtained (22.76) might suggest a medium parasitic load into the sample.

*A. vasorum* was also amplified in cases 2 and 4. In case 2, The BLAST analysis revealed a 99.84% nucleotide identity (610/611 nucleotides) to two *A. vasorum* isolates, originating from red foxes in the UK (Accession numbers GQ982791 and GQ982741). The sequence was deposited in GenBank^®^ under the Accession Number OQ210698. In case 4, The BLAST analysis indicated 100% nucleotide identity to numerous *A. vasorum* isolates (578 nucleotides) from red foxes from the UK (Accession Numbers: GQ982791, GQ982741, LT990053-LT990072). The sequence was deposited in GenBank^®^ under the Accession Number OQ210699.

**Figure 5:**
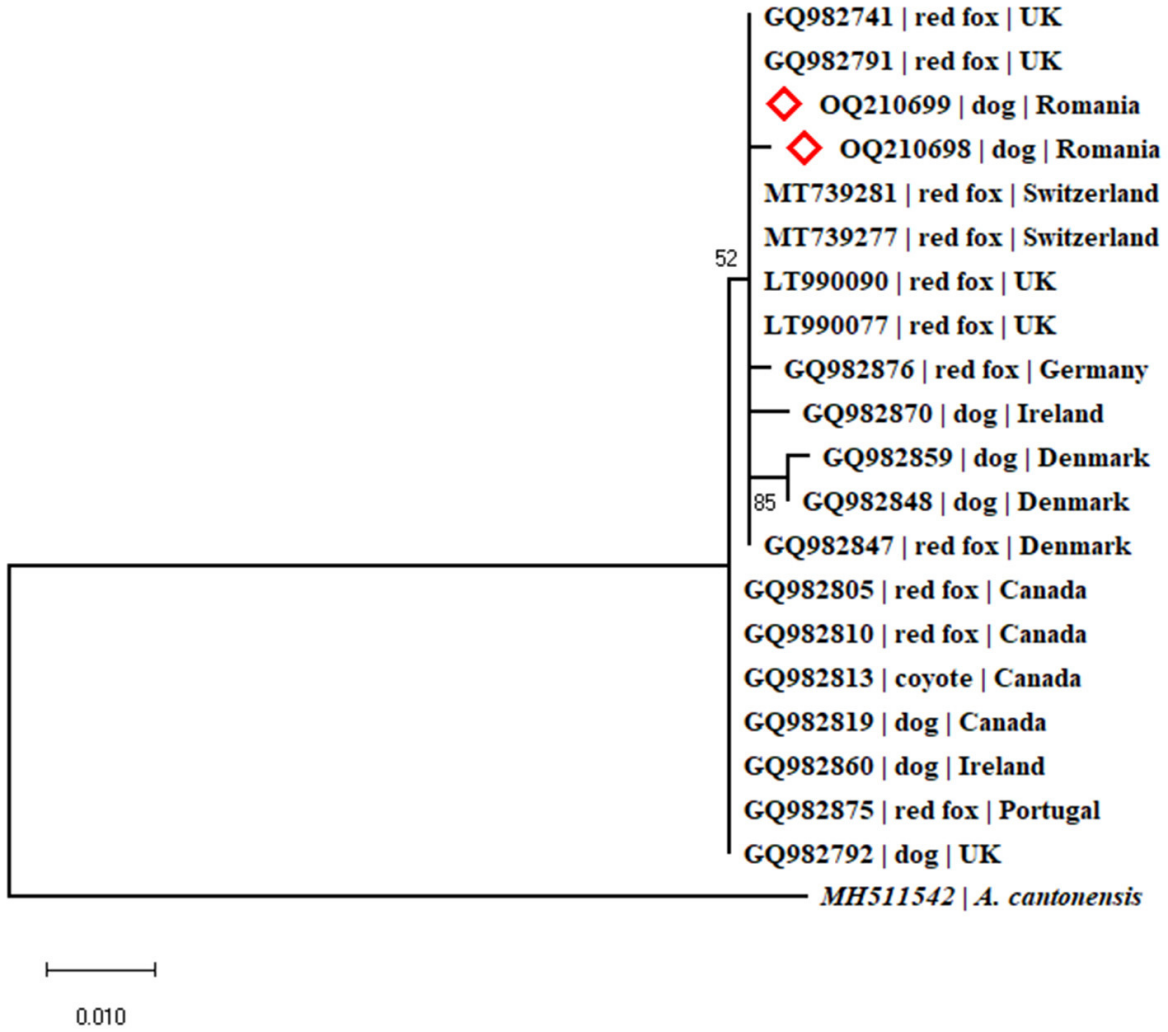
Phylogenetic tree constructed by Maximum Likelihood method and Tamura-Nei model. The tree with the highest log likelihood (-1039.46) is shown. The percentage of trees in which the associated taxa clustered together is shown next to the branches. The tree is drawn to scale, with branch lengths measured in the number of substitutions per site. The analysis involved 21 nucleotide sequences. There were a total of 564 positions in the final dataset.

The phylogenetic analysis was performed using MEGA X software (30), using the Maximum Likelihood method, and Tamura and Nei model (31). Overall the analysis included 20 *A. vasorum* sequences obtained from domestic dogs and wild carnivores, and one *A. cantonensis* sequence as outgroup (Figure 5).

## 4. Discussion

**Figure 6:**
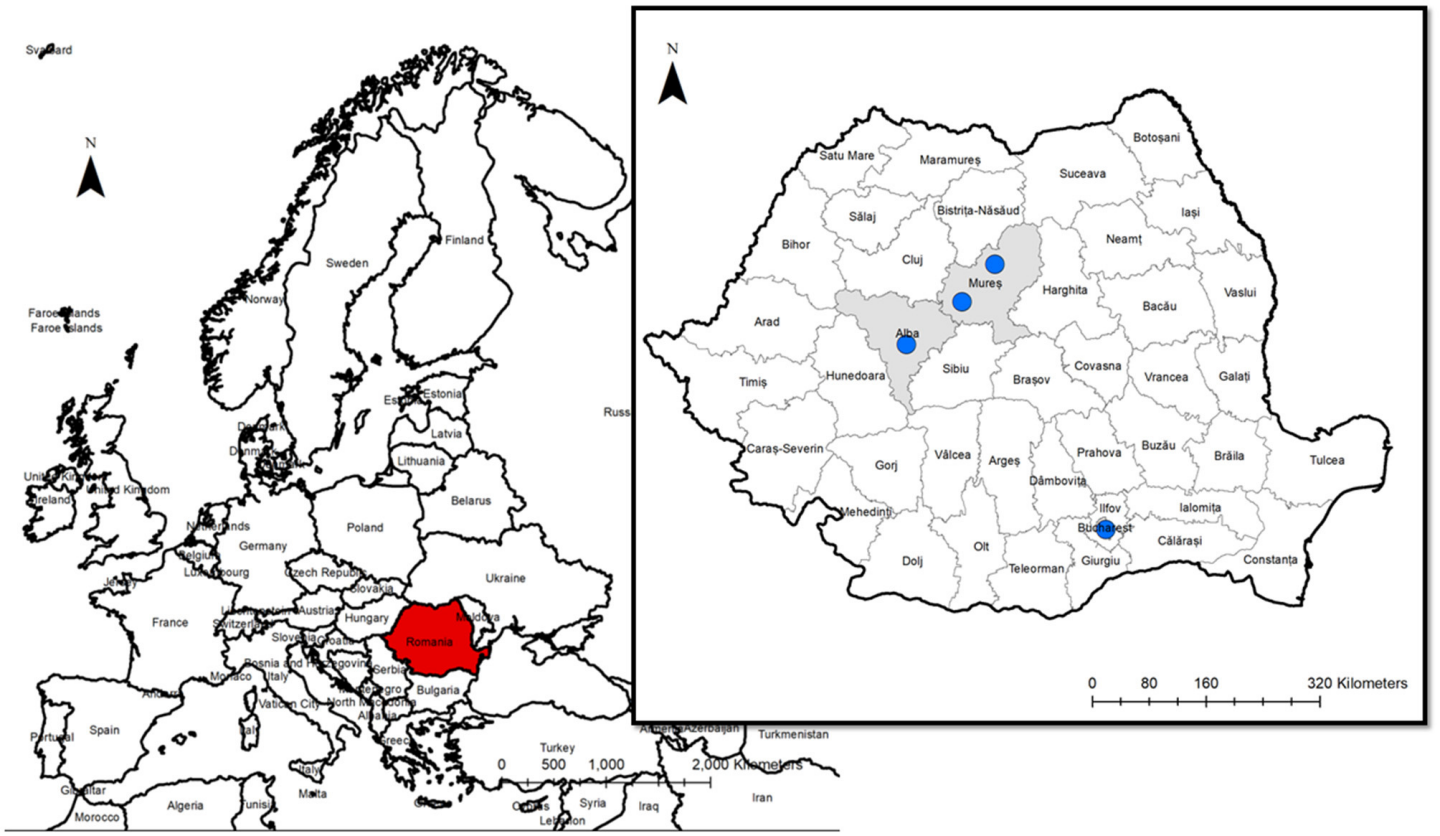
The geographical origin of the dogs infected by *A. vasorum*.

The gastropod-borne nematode *A. vasorum* represents an important cause of cardio-pulmonary disease in dogs worldwide. Although more than 5 years passed since the first identification of *A. vasorum* in Romania, no clinical cases were yet diagnosed. The study aimed to describe the first clinical cases of canine angiostrongylosis in Romania (Figure 6). *Angiostrongylus vasorum*'s distribution in Europe is characterized by hyperendemic foci in the proximity of low prevalence zones. The increase of *A. vasorum* reports in the last years could be due to wildlife movements, domestic dog relocations, and possibly climatic changes associated with an increasing number of gastropods (32, 33).

In a specific study aiming to determine the knowledge about canine angiostrongylosis among veterinarians, only 8.5% of them confirmed to have been diagnosed with a case, with the bulk of them hailing from Bucharest (25). In the same study, a lack of knowledge about this parasitic disease was detected particularly in rural areas where the veterinary focus is still centered mostly on farm animals. Besides a low interest from rural veterinarians in diagnosing and treating pet animals, the second problem is represented by the owners. In this study, only one dog (case 1) was correctly diagnosed intravitam and specific antiparasitic treatment was administered. However, due to the before-mentioned issue, many pet owners have a very low interest in treating their animals (personal observation) as in the present case when the dog died after 6 months due to respiratory distress.

Typically, infection by *A. vasorum* occurs through the consumption of intermediate or paratenic hosts. None of these dogs were seen to consume gastropods, however, the dog presented in case 2 was known to consume raw fish. Although fish are known as paratenic hosts for *A. cantonensis* (34), another species of the same genus, their role in the biology of *A. vasorum* is not known and further studies are needed to investigate the role of fish in the infection with canine angiostrongylosis.

Red foxes (*Vulpes vulpes*) are known as important reservoirs for parasitic diseases, including zoonoses (35). Experimental infections proved that *A. vasorum* can be transferred from foxes to dogs. Moreover, foxes seem to deal very well with infection and do not typically manifest clinical signs (36). In our case, only the dog from case 2 had free access to the environment and we can assume that infected foxes contaminated the pasture. Infection of urban dogs from cases 1, 3, and 4 can be associated with the presence of foxes in the urban environment which is frequently observed in Romania (personal observations).

The misdiagnosis of cases in Romania should not be related to the available diagnostic tests because there are several detection methods that can be easily used for the identification of canine angiostrongylosis, such as the “gold-standard” Baermann s technique, the AngioDetect rapid test (IDEXX Laboratories, Westbrook, Maine, USA) or genetic methods (28). In a study conducted by Bourque et al. (9), 3 out of 7 (42.9%) cases classified postmortem as positive for *A. vasorum* infection by larvae concentration methods, indicating that sometimes coproscopy is insufficient to provide a positive results. Another recent study comparing three methods for detection of *A. vasorum* in foxes showed that the sensitivity of dissection was higher than nested PCR of an 18S rRNA performed on bronchoalveolar lavage fluid, and canine *A. vasorum* antigen detection test (37).

In the present study, the coproparasitological examination was performed in only one case (case 1) suspected of cardiopulmonary angiostrongylosis based on clinical signs. Infection by *A. vasorum* was subsequently confirmed by PCR, using fluid resulting from the Baermann method. Unfortunately, in this case the postmortem examination was not carried out, and pulmonary lesions induced by these nematodes were not evaluated. As demonstrated by the three other lethal misdiagnosed cases (cases 2, 3 and 4), specific diagnosis methods (coproscopy) were not used due to the lack of knowledge related to *A. vasorum* infection in Romania. On the other hand, this scenario may lead to the use of ineffective treatment, endangering the animal's life, as in the described cases. Lack of awareness among veterinarians related to *A. vasorum* and non-specific clinical signs associated with infection (dyspnea, coughing, and exercise intolerance) can further complicate the diagnosis (38). However, angiostrongylosis should be considered as a potential cause in all dogs with respiratory symptoms, especially in those with free access to the environment. Interestingly, although common, no nervous signs were clinically observed in any of these cases. Moreover, as previously demonstrated, *A. vasorum* L1 and inflammatory response were detected in CNS (Case 2). The presence of L1 in the nervous system is considered to be related to neurological disorders (1).

The coagulation profile was available only for one of the dogs (case 1), and there was prolonged PT time, while aPTT time was within the normal reference interval, and platelet numbers were moderately increased, suggesting a disseminated intravascular coagulation (DIC) status. In most cases of canine angiostrongylosis, the platelet count is either within normal limits or more commonly slightly to moderately decreased (39, 40). Petechial hemorrhages associated with bleeding disorders were not recorded by the clinician in this dog. The link between infection by *A. vasorum* and hemostatic dysfunction is not fully elucidated, but the main theories proposed are as follows: (1) widespread immune complex deposition and complement fixation, which would lead to disseminated intravascular coagulation (41); (2) chronic infections may lead to downregulation of numerous proteins involved in homeostasis (42); and (3) hyperfibrinolysis (43).

Gross, cytological and histological changes were similar in all diagnosed cases, and in accordance with previously reported findings (9). Macroscopical findings of the lungs consisted in all cases of multifocal to coalescing dark-red densification areas, associated with hemorrhage and necrosis. The most consistent histological finding was granulomatous interstitial pneumonia associated with the intra-alveolar larvae and eggs. The presence of adult parasites was restricted to medium-sized and large arteries, and often they were embedded in a thrombotic mass and associated with proliferative endarteritis. Even though the main localization of the adult parasites is within the pulmonary arteries, they may be rarely encountered in unusual locations such as the pericardial sac, urinary bladder, kidney, and femoral artery (44–46). Larvae have been identified in numerous tissues, as follows: brain, cerebellum, liver, pancreas, skin, and kidney (44, 47). In this case series, the localization of the adult parasites was restricted to pulmonary arteries but in one case, numerous adult parasites were also identified in the right ventricle. From each case, several tissues were collected, but larvae and secondary inflammatory changes were identified only in the tracheobronchial lymph nodes and brain (cases 2 and 4). In only one case (case 4), nodular gastritis, affecting the body region, was identified. Nodular (follicular) gastritis defines marked mononuclear inflammatory infiltrates and the formation of lymphoid follicles with a germinal center in the lamina propria of the gastric mucosa. In dogs, the cause in still unknown, and some theories including *Helicobacter*-like organism infections and chronic respiratory distress especially in brachycephalic dogs (e.g., French bulldogs) have been proposed (48). In our case, no parasitic organisms were identified in the gastric nodular lesions.

Considering all these aspects, there is a strong need to raise awareness of this parasitosis in domestic canids in Romania among clinical veterinarians and dog owners.

## 5. Conclusions

This study reports the first clinical autochthonous case of infection with *A. vasorum* in a domestic dog from Romania and three other cases of fatal angiostrongylosis diagnosed post-mortem. We can confirm that respiratory signs represent the main clinical picture in infected dogs. Further national large-scale studies are needed for a complete picture of this disease in regard to its prevalence, distribution, biology and diagnostic methods in domestic dogs. Considering the severity of canine angiostrongylosis, national programs for continuous education could help advocate the importance of testing all symptomatic dogs and considering infection with *A.vasorum* as a part of differential diagnosis.

## Data availability statement

The datasets presented in this study can be found in online repositories. The names of the repository/repositories and accession number(s) can be found in the article/Supplementary material.

## Ethics statement

Ethical review and approval was not required for the study on animals in accordance with the local legislation and institutional requirements. Written informed consent was obtained from the owners for the participation of their animals in this study.

## Author contributions

MAT conceived and wrote the manuscript. MAT, CT, and AU performed the postmortem examination and sample prelevation. AN and IS performed the cytological examination. AI and MT performed the molecular work. AN and CG performed the histological examination. GD coordinated the study. GD, AI, and CG critically revised the manuscript. AB, AT, and IH did the clinical investigation and they were responsible for the follow-up. All authors contributed to the article and approved the submitted version.
