# Supplementary material for: Is the Angiostrongylus vasorum infection in domestic dogs underestimated or misdiagnosed? A comprehensive presentation of four lethal cases
Source: Front Vet Sci. 2023 May 18;10:1146713. doi: 10.3389/fvets.2023.1146713 (PMC10232773; doi:10.3389/fvets.2023.1146713)
Supplement: Supplementary file 1 [file Data_Sheet_1.pdf]

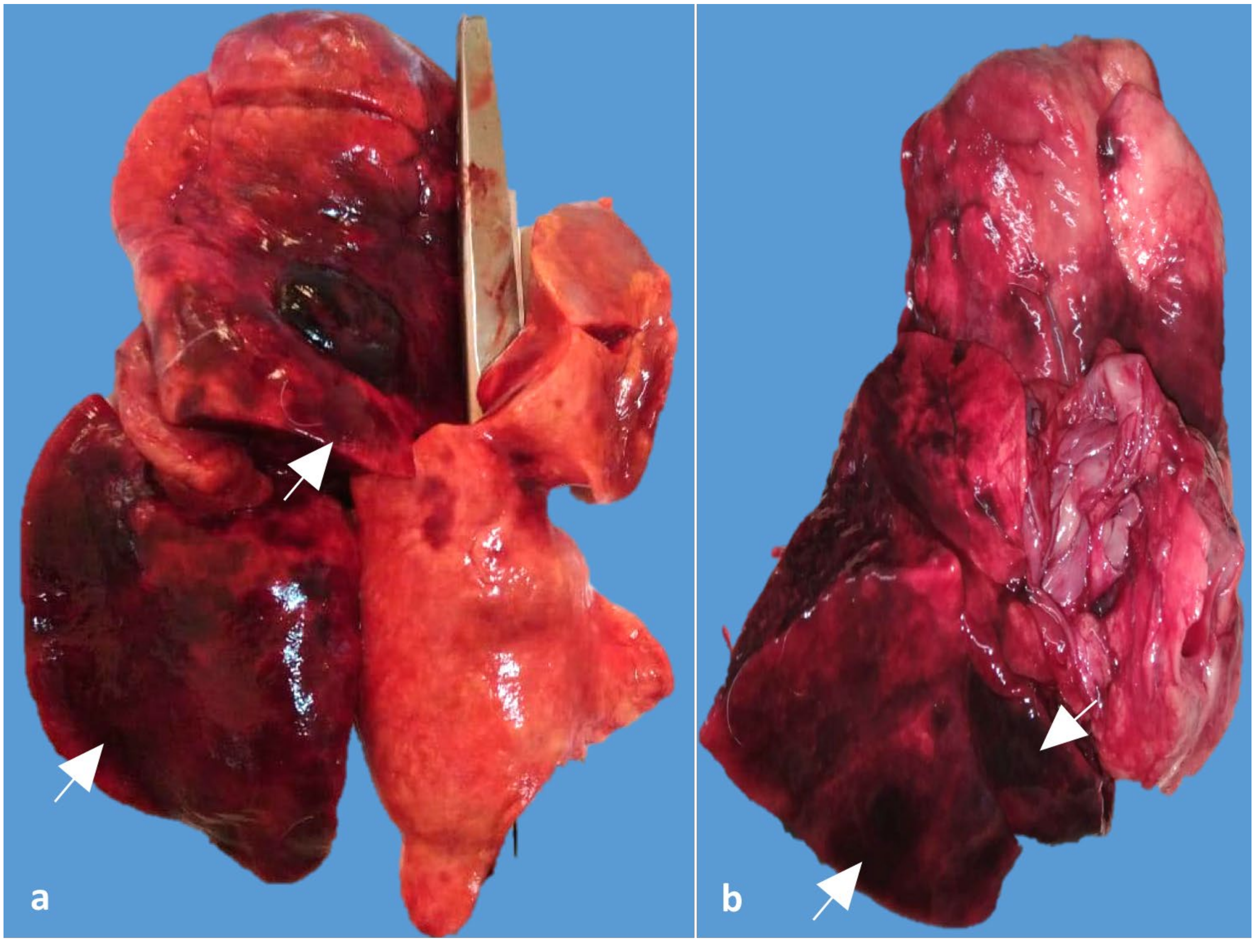

Supplementary Figure 1. Case 3. Gross evaluation of the lungs showing multifocal to coalescing dark red areas of hemorrhage and necrosis (white arrows), a) dorsal view and b) ventral view.

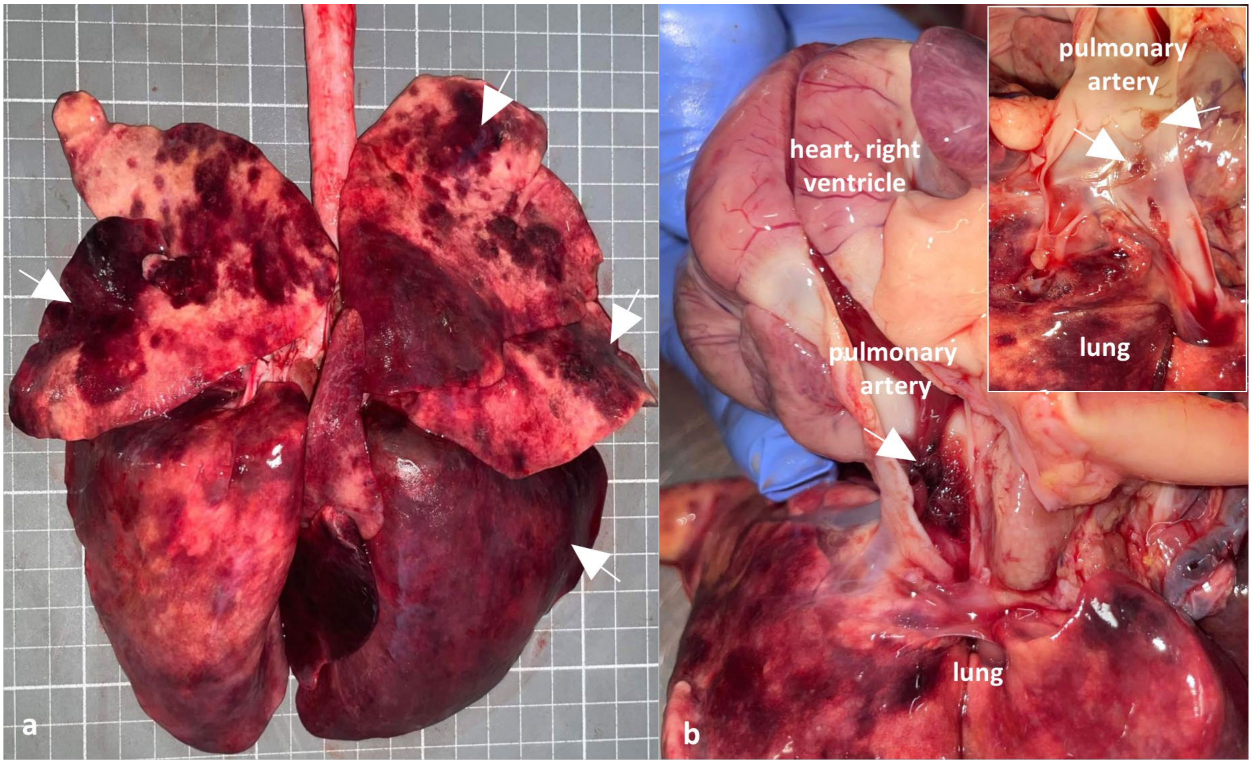

Supplementary Figure 2. Case 4. a) Gross examination of the lungs showing bilateral and multifocal necrosis and hemorrhages (arrows); b) Numerous adults of *Angiostronylus vasorum* in the pulmonary artery (arrows and the inset).

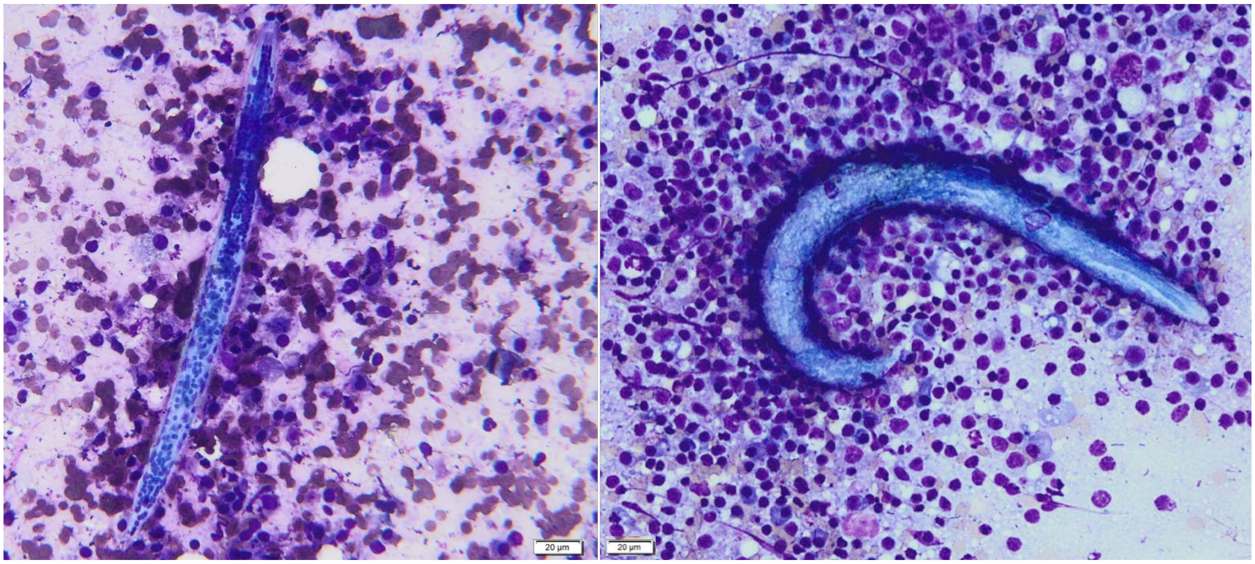

Supplementary Figure 3. Case 2. Cytological exam demonstrating larvae of *A. vasorum* in the lungs (a) and tracheobronchial lymph nodes (b) associated with an inflammatory reaction composed of macrophages and plasma cells. DQP stain. Bar=20 µm.

The sequences registered in the NCBI database:

1) *Angiostrongylus vasorum* isolate Case_2 cytochrome c oxidase subunit I (COX1) gene, partial cds; mitochondrial.

https://www.ncbi.nlm.nih.gov/nuccore/OQ210698

2) *Angiostrongylus vasorum* isolate Case_4 cytochrome c oxidase subunit I (COX1) gene, partial cds; mitochondrial.

https://www.ncbi.nlm.nih.gov/nuccore/OQ210699
